# Identifying canopy wilting QTLs and evaluating remote sensing approaches for selecting drought-tolerant soybean

**DOI:** 10.1007/s00122-025-05063-y

**Published:** 2025-10-14

**Authors:** Nathaniel Burner, Price Pius Atuah Akiina, Qijian Song, Donna K. Harris, Zenglu Li

**Affiliations:** 1 Institute of Plant Breeding, Genetics, and Genomics, and Department of Crop and Soil Sciences, Univ. of Georgia, Athens, GA USA; 2 Department of Plant Sciences, Univ. of Wyoming, Sheridan Research and Extension Center, Sheridan, WY USA; 3 Soybean Genomics and Improvement Laboratory, USDA-ARS, Beltsville, MD USA

## Abstract

Drought is the most damaging abiotic stress for soybean yield; cultivars with improved drought tolerance are needed to sustain and increase crop production. PI 603535 previously was identified as an ultra-slow canopy wilting (CW) line in a genome-wide association study but the quantitative trait loci (QTLs) underlying this phenotype have not been determined. In this study, a recombinant inbred line (RIL) population derived from Benning × PI 603535 was evaluated for three years under rain-fed conditions. CW was rated following extended periods of drought when CW variation was present. Aerial multispectral and thermal imagery was also captured in conjunction with visual ratings to explore the feasibility of implementing remote sensing to improve the efficiency and objectivity of drought evaluations. The normalized difference vegetation index (NDVI) and green-based NDVI (GNDVI) exhibited strong, significant correlations (|r|= 0.42–0.44) with CW across years. CW scores and the remote sensing traits were used as phenotypes for QTL mapping. Seven CW QTLs were identified across six chromosomes in the combined analysis, with NDVI and GNDVI QTLs generally colocalizing with the CW QTLs with the highest percentage of variation explained (PVE). The QTLs were not consistently identified among individual years, highlighting the complex genetics and gene expression of drought tolerance. The instability and low additive effect estimates of individual QTLs imply challenges of improving drought tolerance through the selection of a few QTLs. However, the slow CW RILs developed in this study can serve as valuable breeding stocks for future drought improvement breeding efforts and genetic studies.

**Supplementary Information:**

The online version contains supplementary material available at 10.1007/s00122-025-05063-y.

## Introduction

Drought is the most damaging abiotic stress for soybean [*Glycine max* (L.) Merr.], reducing yields by approximately 40% and results in billions of dollars in insurance indemnities annually (Boyer 1982; Specht et al. 1999; Wilhite et al. 2007). Drought stress occurs when the demands of transpiration and evapotranspiration exceed the availability of water (Rauf et al. 2016). This is a particular problem for soybean as only a fraction of harvested acres is irrigated (USDA 2024).

Major breeding efforts to improve soybean drought tolerance in the USA did not begin until the early 1990s due to challenges such as skepticism of yield potential, lack of genetic variability for drought tolerance among modern soybean lines, and inconsistent drought conditions across environments (Brown et al. 1985; Carter, 1999, Carte et al. 2004; Gizlice et al. 1994; Sneller & Dombek 1997). PI 416937 is the earliest identified slow CW accession that has been used by southern soybean breeding programs to develop varieties with improved drought tolerance and competitive yields (Carter, 1999). ‘N7001’ was the first publicly released US soybean cultivar to feature PI 416937 in its pedigree and subsequently has been used as a genetic stock in the development of other notable cultivars such as ‘N7002’, ‘N7006’, ‘N8001’, and ‘Woodruff’ (Boerma et al. 2012; Carter & Rufty  1993; Carter. et al. 2007, 2008; Fallen et al. 2023). Further, the inclusion of exotic germplasm is attractive to soybean breeders as a means of broadening the very narrow genetic base of the soybean germplasm (Carter et al. 2004; Gizlice et al. 1994).

Drought tolerance is a highly polygenic trait as evident by the many combinations of physiological mechanisms that have been observed to influence it (Purcell & Specht 2004; Ludlow & Muchow 1990; Ries et al. 2012; Valliyodan et al. 2017; Menke et al. 2024). QTL mapping using RIL populations derived from crosses between parents of differing relative drought sensitivities has been the primary method for identifying genomic regions associated with drought tolerance-related traits such as CW, fibrous roots, and canopy temperature (Abdel-Haleem et al. 2011, 2012; Bazzer & Purcell 2020; Charlson et al. 2009; Du et al. 2009; Hwang et al. 2015; Menke et al. 2024). Genome-wide association (GWAS) studies have recently been used to identify genetic variability associated with the CW trait without the need for population development (Kaler et al. 2017; Steketee et al. 2020). Generally, some degree of consistency has been observed regarding commonly detected regions in the QTL mapping and GWAS studies. For example, nine of the 45 detected SNPs in the GWAS study conducted by Steketee et al. (2020) were located in regions previously detected in the CW mapping studies (Abdel-Haleem et al. 2012; Charlson et al. 2009; Hwang et al. 2015). Most detected QTLs in these studies have not conclusively been associated with specific genes and the associated genetic variability, but they have been hypothesized to be colocalized with gene models for agronomic traits and stress responses (Abdel-Haleem et al. 2012; Charlson et al. 2009; Kaler et al. 2017; Steketee et al. 2020).

A precise phenotyping is required to accurately associate genomic regions with drought tolerance traits. However, traditional phenotyping of drought tolerance requires subjective visual ratings (CW), destructive phenotyping (root architecture), or time-sensitive leaf tissue sampling (transpiration rate, N_2_ fixation, stomatal conductance, carbon isotope discrimination, etc.) (Hudak & Patterson 1995; King et al. 2009; de Paula et al. 2024; Steketee et al. 2019; Tanaka et al. 2010). Evaluations of physiological processes related to drought are often done in greenhouses or growth chambers using the seedlings due to the variability posed by inconsistent environmental conditions (Riar et al. 2018; Steketee et al. 2019; Wang et al. 2020). Kwon et al. (2025) identified a major QTL on Chr 4 using pot weight measurements to predict transpiration rates during the early vegetative stage in greenhouse. However, these greenhouse results may not represent drought tolerance under field conditions at later growth stages. Remote sensing using unmanned aerial vehicles (UAVs) has been used extensively in plant breeding and agronomic research to associate spectral metrics with physiological and morphological traits that may be otherwise difficult to phenotype (Guo et al. 2021). Remote sensing has been used in soybean drought research to make phenotyping more efficient, objective, and standardized. Bai and Purcell (2018) used aerial infrared imagery to identify a positive relationship between relative canopy temperature (CT) and drought stress. Further, slow CW genotypes exhibited reduced CT compared to fast wilting genotypes even on dates without visual CW differences. This study observed a consistent negative correlation between CT and yield; however, the authors emphasize that further validation of this association is required. Zhou et al. (2020) developed a support vector machine model consisting of remote sensing traits (NDVI, GNDVI, CT, hue, and saturation) and morphological characteristics (canopy size and height) to distinguish between fast and slow CW soybean lines with an average accuracy of 80%. CT and NDVI specifically were found to be significantly different between wilting classes. These studies indicated that remote sensing traits have the potential to serve as selection criteria for drought tolerance.

CT has been used as a proxy metric for drought tolerance due to its association with the evaporative cooling effect of increased stomatal conductance and water access. Kaler et al. (2018) identified eight and 52 SNPs associated with CT in an association mapping study of individual and combined environments, respectively. Approximately half of these SNPs were located directly in genes with functions relating to root architecture, stress signaling, and stomatal complex morphogenesis. Additionally, 15 genomic regions coincided with those for CW identified by Kaler et al. (2017). Bazzer and Purcell (2020) performed composite and multiple interval mapping for CT in a ‘KS4895’ × ‘Jackson’ RIL population and identified 11 loci on eight chromosomes on which QTLs for CW, CT, carbon, and oxygen isotope discrimination, and water use efficiency were previously mapped. These results offer promising evidence that remote sensing can provide accurate phenotypic information to improve the efficiency and accuracy of breeding decisions and aid the identification of putative causal genomic regions associated with drought tolerance.

PI 603535 is a MG VIII accession that was identified amongst a diverse panel of MG VI-VIII accessions from the USDA Soybean Germplasm Collection that exhibited the lowest CW rating in a multiple year and location genome-wide association study (Steketee et al. 2020). The CW score of PI 603535 was lower than PI 416937 in every environment tested. The traits or genes conferring the slow CW phenotype have not yet been characterized due to the recency of its identification. Nevertheless, PI 603535 could be a useful source of genetic variation in the development of drought-tolerant soybean cultivars. Identifying the QTLs and developing genetic markers in genomic regions associated with slow CW will improve the efficiency of breeding efforts. Further, drought mapping studies in soybean using remote sensing phenotypes have been limited to canopy temperature. To our knowledge, there have been no mapping studies for soybean drought tolerance utilizing multispectral-derived indices. Like canopy temperature, multispectral indices may also be useful for high-throughput phenotyping of drought tolerance and identifying genomic regions associated with it. The objectives of this study were to 1) identify genomic regions associated with the slow CW phenotype of PI 603535 and 2) assess the feasibility of using remote sensing traits for drought tolerance phenotyping and genetic mapping of drought tolerance traits.

## Materials and methods

### Plant materials

A recombinant inbred line (RIL) population was developed from a cross derived from Benning × PI 603535. Benning is an elite MG VII cultivar developed at the University of Georgia (Boerma et al. 1997). PI 603535 is a MG VIII accession identified by Steketee et al. (2020) among 162 MG VI-VIII accessions from the USDA Soybean Germplasm Collection that exhibited the lowest CW score across a multiple year and location evaluation for a genome-wide association study. The progeny of this cross was advanced to the F_5_ generation by single-seed descent where individual plants were harvested to obtain seed for 188 F_5_-derived RILs.

A total of 188 RILs, both parents and five checks, were grown for three years during the 2022–2024 growing season in Wyoming. In 2022, the experiment was grown in Sheridan, Wyoming (44°45′38"N 106°56′27"W), and in 2023–2024 it was grown at Wyarno, WY (44°50′25″N, 106°50′20″W), which is approximately 14 km from the Sheridan location. The soil at both sites is predominantly Wyarno clay loam (O’Geen et al. 2017). The experiment was in a randomized complete block design consisting of three replicates. These locations were used because this area historically receives very little rainfall during the growing season and could therefore provide relatively consistent drought conditions over the course of the study. Plots were planted as two-row plots with 0.76-m row spacing and 3.05 m length at a planting density of approximately 43 seed m^−2^.

In 2022, according to the National Weather Service for Sheridan County Airport, approximately 4 km from the field site, the site received 61 mm of rainfall. In 2023 and 2024, the weather station located at the farm in Wyarno, WY, showed that the site received, 216- and 88-mm rainfall, respectively, between planting and CW ratings. In the 30 days prior to CW ratings, the sites received 3.6, 44, and 19 mm of rain in 2022–2024, respectively. In 2022, the site received 57.4 and 61 mm of rain between planting and the CT and multispectral measurement dates, respectively. In the 30 days prior to these dates, the site received 14.2 and 14.0 mm, respectively.

### Evaluation of canopy wilting and remote sensing traits

CW was visually scored on a scale of 0 to 100 in increments of five as described by King et al. (2009), with higher scores indicating greater wilting severity. Ratings were performed by either a single (2022–2023) or multiple (2024) raters multiple times between the establishment of full canopy coverage and R2 growth stage (Fehr et al. 1971).

Multispectral and thermal imagery was captured concurrently or within a few days of CW ratings. In 2022, two handheld devices, a RapidScan CS-45 (Holland Scientific, Lincoln, NE) and Apogee MI-2H0 (Apogee Instruments, Logan, UT), were used to measure canopy NDVI and temperature (CT), respectively. In 2023 and 2024, multispectral and thermal imagery was captured with a DJI Mavic 3M and a DJI Mavic 3 Thermal (SZ DJI Technology Co, Shenzhen, China), respectively, from approximately 25 m above ground with 85% front and side overlap near solar noon.

Pix4Dmapper (v4.6.4, Pix4D, Prilly, Switzerland) was used to generate multispectral and thermal orthomosaics using the ‘Ag Multispectral’ and ‘Thermal camera’ processing templates. Reflectance maps were generated for the vegetative indices (VIs), normalized difference vegetation index (NDVI) (Rouse et al. 1974), normalized difference red edge index (NDRE) (Barnes et al. 2000), and green-based NDVI (GNDVI) (Sripada et al. 2006). These VIs are popular in remote sensing applications for monitoring general plant health. NDVI, GNDVI, and NDRE are expressed on a scale from -1 to 1 and reflect the amount of red, green, and red edge reflectance relative to near-infrared (NIR) reflectance. Positive values generally indicate the presence of vegetation, with higher values associated with healthier vegetative tissue and greater density.

Orthomosaics were loaded into QGIS (v3 + , QGIS Development Team) for visualization, and plot shapefiles were generated using the plugin ‘SHP Buddy’ (Burner et al. 2025). A vegetation mask layer was generated with the ‘raster calculator’ processing function using an excess green index threshold of 0.08 (Woebbecke et al. 1995). This threshold was determined by visually inspecting that the masked canopy outlines corresponded to the true canopy outlines in the RGB raster. The mean NDVI, NDRE, GNDVI, and CT values for each plot were calculated using the ‘zonal statistics’ processing function in QGIS. CT values were normalized (nCT) within each date using a min–max normalization according to Kaler et al. (2018) to account for differential ambient temperatures. nCT values within each year are expressed on a scale of 0 to 1, with higher values indicating warmer relative CTs.

### Statistical analysis

Analyses of variance (ANOVA) for single year and combined years were performed for CW and the remote sensing traits using PROC GLM in SAS Studio (SAS Institute, Cary, NC). Least squares means (Lsmeans) for each genotype were separated using the Tukey–Kramer test (α = 0.05, Kramer 1956). Best-linear unbiased predictions (BLUPs) were calculated for each genotype within and across years with PROC MIXED using a model that treated genotype, year, genotype × year, and replicate nested within year as random variables. BLUPs were then used as phenotypic values for downstream QTL analyses.

The 2022 trait values were from Aug 19, Aug 29, and Sept 7 for nCT, NDVI, and CW, respectively. The values for all traits in 2023 and 2024 were from Sept 3. Different dates in 2022 were used because NDVI and nCT exhibited strong field effects on dates closer to the CW date. An additional combined analysis consisting of only the remote sensing traits collected in 2023–2024 was used to account for potential differences caused by the different phenotyping equipment. In the combined analyses, the genotype × year term was used as the denominator for testing of genotype and year effects. Lastly, broad-sense heritability was calculated on an entry-mean basis using variances derived from each model (Holland et al. 2003).

### Genotyping

Leaf tissue collection and DNA extraction from RILs were performed as described by Steketee et al. (2020). DNA samples from each RIL were genotyped using the SoySNP6K iSelect BeadChip (Song et al. 2013). Genome Studio (v 2.0 + , Illumina, San Diego, CA) was used to call SNP genotypes and quality control. SNP data for Benning and PI 603535 was downloaded from Soybase (Grant et al. 2010). RIL SNP calls were coded to match the corresponding parental genotypes whereby ‘A’ corresponded to the Benning allele and ‘B’ to the PI 603535 allele. Missing marker genotypes were imputed in R/qtl using the ‘Kosambi’ method (Broman & Sen 2009). Markers that were monomorphic or exhibited segregation distortion were removed. After quality control, 1,373 markers remained in the marker data set for QTL mapping.

### Genetic map construction and QTL analysis

The R package ‘ASMap’ was used to construct a genetic linkage map using the ‘Kosambi’ function (Taylor & Butler 2017). A *p*-value of 0.01 was used to place the markers into the expected 20 linkage groups in soybean. A heatmap of the recombination fractions was visually inspected for markers with problematic linkage patterns or marker orders and were resolved manually either through exclusion or forcing marker orders.

QTL mapping using the BLUP values for CW, NDVI, NDRE, GNDVI, and nCT was performed for within and across years using R/qtl (Arends et al. 2010; Broman & Sen 2009). The combined analysis used CW, NDVI, and nCT from 2022 to 2024 and GNDVI and NDRE from 2023 to 2024. Cofactors were initially set every 10 markers using the *mqmsetcofactors* function. Uninformative cofactors were removed through backwards elimination during the whole-genome scan. Whole-genome scans were performed using the *mqmscan* function with a step size of 1.0 and cofactor significance of α = 0.05. The locations of QTLs exhibiting logarithm of odds (LOD) scores > 3 were used as main effects in the *fitqtl* function to obtain estimates of additive effects and percent variation explained (PVE).

### Candidate gene identification

From the combined analysis, CW QTLs that colocalized with remote sensing QTLs were used to identify candidate genes. Further, the largest remote sensing QTLs by PVE were also used to identify candidate genes. A 10-kb region flanking the Wm82.a4 position of the nearest marker to each QTL was scanned for candidate genes using the Glyma4.1 gene models in SoyBase (Grant et al. 2010; Song et al. 2024).

## Results

### Phenotypic variation through visual rating

**Fig. 1:**
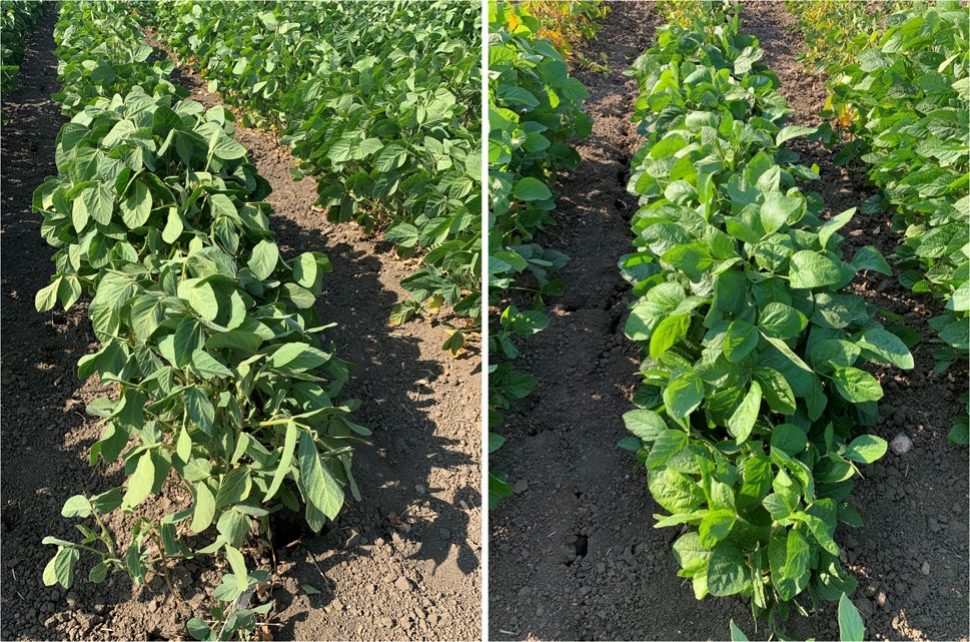
Fast wilting parent Benning (left) and slow wilting parent PI 603535 (right)

**Fig. 2:**
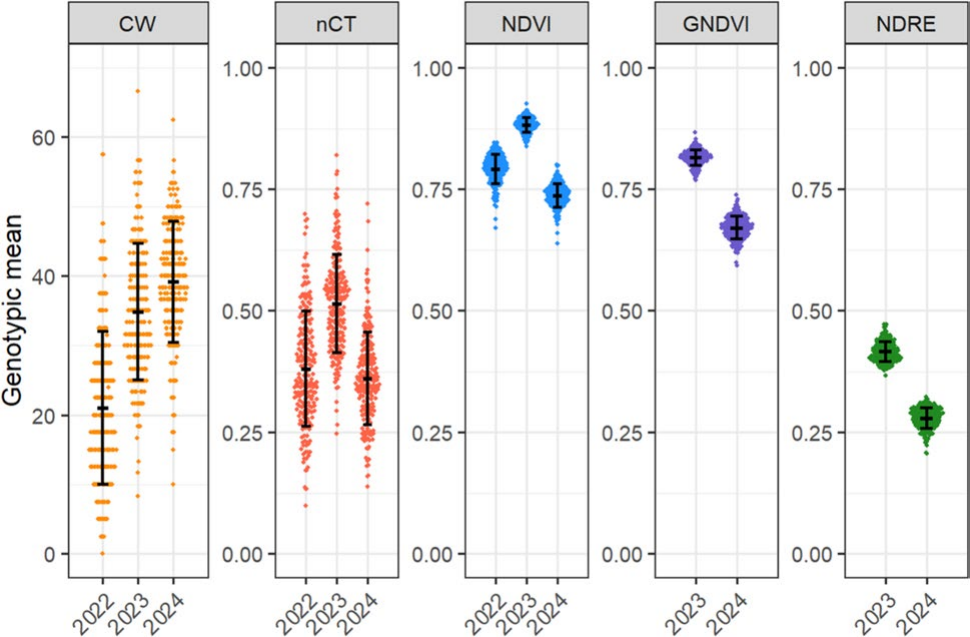
Distribution of genotypic means for CW scores and nCT, NDVI, GNDVI, and NDRE measurements. nCT is the relative CT among genotypes within each year and NDVI, GNDVI, and NDRE indicate the proportion of NIR reflectance relative to red, green, and red edge reflectance, respectively, with higher values indicating healthier and denser canopies. Error bars indicate the mean and standard deviation for each trait × year combination. 2022 remote sensing traits were collected with handheld devices, whereas 2023–2024 was collected from UAVs (CW = Canopy wilting; nCT = Normalized canopy temperature; NDVI = Normalized difference vegetation index; GNDVI = Green-based normalized difference vegetation index; NDRE = Normalized difference red edge index)

The drought conditions resulted in wide ranges of values for all traits in each year (Figs. 1and2, Table 1). Lsmeans for CW ranged from 5.7 to 58.1 in the combined year analysis and the effects of year, genotype, and genotype × year were statistically significant for CW (Supplemental Table 1). Further, the effects of genotypes were significant in each individual year analysis. The average coefficients of variation (CV) of genotypic CW within each year ranged from 29.9 to 52.3%. CW heritability was high (*H*^*2*^ = 0.70) across years and ranged from 0.50 to 0.61 in individual years (Supplemental Table 1). All RILs exhibited CW scores between PI 603535 and Benning (Fig. 2, Table 1). PI 603535 exhibited the lowest CW score in all individual years except for 2022 in which 12 RILs exhibited CW scores of equal or lesser value. Across the years, five RILs exhibited CW scores of < 20.

**Table 1:** Performance and major QTL genotypes of 10 RILs with highest and lowest CW scores, along with the parental genotypes from combined analysis.

Type	Genotype	CW	nCT	NDVI	GNDVI	NDRE	*qWilt_Gm07*	*qWilt_Gm12*	*qWilt_Gm13*	*qNCT_Gm17*	*qNDVI_Gm13.1*	*qGNDVI_Gm12*	*qNDRE_Gm02*
Parents	PI 603535	5.7	0.50	0.81	0.75	0.32	PI	PI	PI	PI	PI	PI	PI
	Benning	58.1	0.53	0.77	0.72	0.35							
Slow wilting	G21-2157	13.6	0.28	0.85	0.80	0.39	PI	PI	PI	PI		PI	PI
	G21-2318	16.5	0.42	0.84	0.78	0.36	PI	PI	PI	PI	–	PI	PI
	G21-2305	18.6	0.49	0.81	0.75	0.34	PI	PI	PI	PI		PI	
	G21-2322	19.2	0.51	0.81	0.74	0.34		PI	–	PI		PI	
	G21-2313	19.4	0.40	0.81	0.75	0.34		PI	H			PI	PI
	G21-2175	20.6	0.30	0.82	0.76	0.36			–		PI		
	G21-2273	20.8	0.46	0.81	0.75	0.34	PI	PI	PI			PI	PI
	G21-2230	21.3	0.44	0.79	0.73	0.34		PI	PI			PI	PI
	G21-2337	21.6	0.30	0.81	0.74	0.35	PI	PI		PI		PI	PI
	G21-2302	22.2	0.45	0.81	0.75	0.34	PI	PI	PI	PI		PI	
Fast wilting	G21-2271	43.1	0.41	0.80	0.71	0.33		–	PI		–		PI
	G21-2179	43.6	0.51	0.77	0.70	0.33		PI					
	G21-2253	43.9	0.43	0.77	0.73	0.35	PI			PI			
	G21-2270	44.1	0.32	0.83	0.77	0.38	PI	PI			PI	PI	PI
	G21-2191	44.2	0.43	0.76	0.74	0.35				PI			
	G21-2168	45.8	0.31	0.79	0.74	0.36	PI			PI			
	G21-2292	46.9	0.40	0.80	0.74	0.35				PI	PI		PI
	G21-2340	47.8	0.41	0.79	0.71	0.33					H		
	G21-2334	48.1	0.45	0.77	0.72	0.33				PI			
	G21-2245	53.1	0.51	0.77	0.72	0.34			PI				

PI,  homozygous for PI 603535 allele; H,  heterozygous;—, missing. Homozygous Benning genotypes indicated by blank cells for ease of visualization

CW,  Canopy wilting; nCT,  Normalized canopy temperature; NDVI,  Normalized difference vegetation index; GNDVI,  Green-based normalized difference vegetation index; NDRE,  Normalized difference red edge index

### Phenotypic variation through remote sensing

Similar to CW, the remote sensing traits also exhibited considerable variation in all years (Fig. 2). In the three-year combined analysis, Lsmeans for nCT and NDVI ranged from 0.19–0.60 and 0.76–0.85, respectively. When considering only the 2023–2024 UAV measurements, nCT, NDVI, GNDVI, and NDRE Lsmeans ranged from 0.20–0.71, 0.76–0.86, 0.70–0.80, and 0.31–0.39, respectively. Effects of year, genotype, and genotype × year were statistically significant for NDVI and GNDVI in the combined analyses (Supplemental Table 1). For NDRE, only genotype and genotype × year were significant in the combined analysis. Genotypic effects were significant for all multispectral traits (NDVI, GNDVI, and NDRE) in all individual year analyses. Among the nCT models, genotype was significant only in the 2-year UAV analysis and 2024. The average genotypic CVs for NDVI, GNDVI, NDRE, and nCT ranged from 2.7–3.9, 2.7–3.6, 8.1–8.9, and 28.5–44.5% among years, respectively.

The remote sensing traits exhibited moderate-to-high broad-sense heritabilities in the combined analyses (Supplemental Table 1). GNDVI exhibited the same heritability (*H*^*2*^ = 0.68) as CW in the combined analysis. Among individual years, NDVI exhibited the highest heritability in 2022 (*H*^*2*^ = 0.60) and GNDVI exhibited the highest heritability in 2023 (*H*^*2*^ = 0.52) and 2024 (*H*^*2*^ = 0.51).

### Correlation between visual ratings and remote sensing traits

**Fig. 3:**
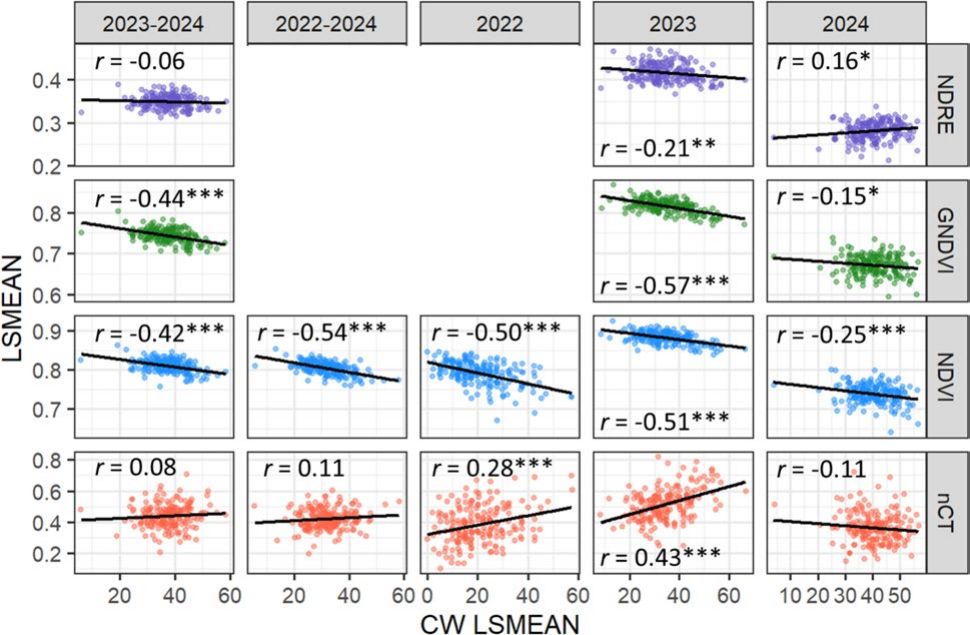
Correlations between CW and remote sensing (nCT, NDVI, GNDVI, NDRE) traits Lsmeans for single years and combined years. GNDVI and NDRE were not available during 2022, hence separate combined plots. Fitted regression line shown in blue. Pearson correlation coefficient and significance indicated for each pair. ***, **, and * indicate *p* ≤ 0.001, 0.01, and 0.05, respectively (CW = Canopy wilting; nCT = Normalized canopy temperature; NDVI = Normalized difference vegetation index; GNDVI = Green-based normalized difference vegetation index; NDRE = Normalized difference red edge index)

The remote sensing traits were generally significantly correlated with CW in individual years, with the exception of nCT in 2024 (Fig. 3). The strongest correlations for all traits occurred in 2023, whereas the weakest occurred in 2024. NDVI and GNDVI exhibited a significantly negative correlation with CW in the combined and single-year analyses. NDRE was significantly correlated with CW only in 2024. In contrast, nCT was significantly positively correlated with CW in 2022 and 2023 but not 2024. NDVI was consistently strongly correlated with CW in individual years (*r* = − 0.25 to − 0.51). Over the years, NDVI and GNDVI were significantly negatively correlated with CW. NDVI and nCT exhibited numerically stronger correlations in the combined analysis (2023–2024) that only evaluated UAV measurements. Within individual years, all remote sensing traits were significantly correlated with each other. This correlation was positive amongst the multispectral traits (NDVI, GNDVI, and NDRE), whereas the correlation between the multispectral traits and nCT was negative. Of these correlations within years, the correlation between NDVI and GNDVI was the strongest (*r* = 0.93) and the correlation between NDRE and nCT was the weakest (*r* = -0.37 to -0.49).

### Canopy wilting and remote sensing QTL analyses

Seven significant QTLs were identified for CW in the combined analysis on Chr 2 (2 QTLs), 3, 7, 12, 13, and 19 (Table 2, Supplemental Fig. 1). PI 603535 contributed the favorable allele (lower CW score) in six of the QTLs with additive effects ranging from -0.79 to -1.42. The PVE for these QTLs ranged from 3.2 to 10.3%, with *qWilt_Gm12* accounting for the largest PVE. A variable number of CW were identified within single years, ranging from only one in 2022 to eight in 2023 (Supplemental Table 2). Chr 12 was the only chromosome on which CW QTLs in similar positions were identified in all three single-year analyses. Of the combined year CW QTLs, only *qWilt_Gm02.1* and *qWilt_Gm13* were identified in multiple individual environments.

**Table 2:** CW and remote sensing QTLs identified by QTL mapping using BLUPs as phenotype values from combined analysis (2022–2024 for CW, nCT, NDVI; 2023–2024 for GNDVI, NDRE)

Trait	Chr^a^	QTL	Nearest SNP	Pos (bp)^b^	Pos (cM)^c^	CI (cM)^d^	LOD^e^	PVE^f^	Effect^g^	+ Allele^h^
CW	2	*qWilt_Gm02.1*	Gm02_43777700	42,533,579	240	235–244	7.4	5.2	− 0.99	PI
	2	*qWilt_Gm02.2*	Gm02_48702118	47,447,513	319	311–320	3.3	3.2	0.84	B
	3	*qWilt_Gm03*	Gm03_41135466	40,295,411	180	170–186	5.7	7.4	− 1.20	PI
	**7**	***qWilt_Gm07***	**Gm07_37260926**	**37,488,675**	**149**	**142–157**	**3.3**	**3.4**	**− 0.79**	**PI**
	**12**	***qWilt_Gm12***	**Gm12_35626081**	**37,053,933**	**233**	**223–239**	**5.3**	**10.3**	**− 1.42**	**PI**
	**13**	***qWilt_Gm13***	**Gm13_37988956**	**38,538,394**	**233**	**229.4–234.4**	**7.7**	**7.6**	**− 1.25**	**PI**
	19	*qWilt_Gm19*	Gm19_823640	855,537	13	5–33	4.1	6.8	− 1.08	PI
nCT	7	*qNCT_Gm07*	Gm07_7284474	7,175,818	71	66–81	3.1	3.9	1.82E-03	B
	11	*qNCT_Gm11*	Gm11_1620921	1,624,684	15	0–26	3.1	2.7	1.61E-03	B
	17	*qNCT_Gm17*	Gm17_15728938	15,449,724	142	136–146.5	3.3	8.8	− 2.55E-03	PI
NDVI	4	*qNDVI_Gm04*	Gm04_9099671	9,157,308	73	66–79	3.7	2.1	9.61E-04	PI
	7	*qNDVI_Gm07.1*	Gm07_1382983	1,386,527	11	3–19	4.7	1.2	7.19E-04	PI
	**7**	***qNDVI_Gm07.2***	**Gm07_37260926**	**37,488,675**	**149**	**146–154**	**4.7**	**2.7**	**1.13E− 03**	**PI**
	**12**	***qNDVI_Gm12***	**Gm12_35876375**	**37,304,589**	**231**	**208–243**	**4.2**	**6.0**	**1.62E− 03**	**PI**
	13	*qNDVI_Gm13.1*	Gm13_579178	20,310,496	71	53–74	5.1	10.0	2.13E-03	PI
	**13**	***qNDVI_Gm13.2***	**Gm13_37144714**	**37,687,029**	**229**	**227–233**	**5.5**	**8.8**	**1.99E− 03**	**PI**
	16	*qNDVI_Gm16*	Gm16_33549185	34,263,137	156	133.1–171	4.2	1.9	9.71E-04	PI
	17	*qNDVI_Gm17*	Gm17_11272874	10,978,068	108	94–122	3.1	3.6	1.27E-03	PI
GNDVI	1	*qGNDVI_Gm01*	Gm01_5453015	5,457,607	86	62–95	4.0	3.3	1.54E-03	PI
	2	*qGNDVI_Gm02*	Gm02_496816	496,539	19	8–24	5.7	5.0	1.87E-03	PI
	7	*qGNDVI_Gm07.1*	Gm07_1382983	1,386,527	12	3–20	3.8	2.6	1.34E-03	PI
	**7**	***qGNDVI_Gm07.2***	**Gm07_37260926**	**37,488,675**	**147**	**129–153**	**3.8**	**3.0**	**1.46E− 03**	**PI**
	12	*qGNDVI_Gm12*	Gm12_34102452	35,530,221	211	208–214	5.7	13.7	3.11E-03	PI
	13	*qGNDVI_Gm13.1*	Gm13_674370	20,215,302	70	50–74	4.6	2.0	1.19E-03	PI
	13	*qGNDVI_Gm13.2*	Gm13_39978113	40,528,851	263	240–269	9.5	6.5	2.21E-03	PI
	17	*qGNDVI_Gm17*	Gm17_15728938	15,449,724	141	136–151	5.9	6.5	2.15E-03	PI
	18	*qGNDVI_Gm18*	Gm18_61009028	56,982,965	289	270–296.9	4.1	3.0	1.51E-03	PI
NDRE	2	*qNDRE_Gm02*	Gm02_4159788	4,192,748	54	11–59	3.7	7.0	2.64E-04	PI
	17	*qNDRE_Gm17*	Gm17_2459036	2,451,384	13	6–16	3.9	6.1	− 2.51E-04	B

Bold QTL indicate colocalization between CW and remote sensing QTL(s)

CW,  Canopy wilting; nCT,  Normalized canopy temperature; NDVI,  Normalized difference vegetation index; GNDVI,  Green-based normalized difference vegetation index; NDRE,  Normalized difference red edge index

^a^Chromosome

^b^Glyma.Wm82.a4 physical position in base pairs (Song et al. 2024)

^c^Genetic map position in centiMorgans

^d^1.0-LOD confidence interval

^e^Log_10_ likelihood ratio comparing full model with reduced model (term dropped)

^f^Percent variation explained

^g^Additive allelic effect

^h^Source of the allele with favorable effect. B,  Benning, PI,  PI 603535. Negative effects for CW and nCT and positive effects for NDVI, GNDVI, and NDRE were considered favorable

The combined analysis identified 3, 8, 9, and 2 significant QTLs for nCT, NDVI, GNDVI, and NDRE, respectively (Table 2, Supplemental Fig. 1). Among the single-year analyses, remote sensing QTLs were only identified in 2023 and 2024 when UAV imagery was used (Supplemental Table 2). The nCT QTLs identified in the combined analysis accounted for between 2.9 and 8.8% PVE, the largest being on Chr 17 (*qNCT_Gm17*) and was the only nCT QTL identified in multiple individual years. This was the only nCT QTL in which the PI 603535 allele was associated with cooler CT. The PVE of the combined NDVI QTLs ranged from 1.2 to 10%. The two largest NDVI QTLs (*qNDVI_Gm13.1* and *qNDVI_Gm13.2*) were identified on opposite ends of Chr 13. Of these, only *qNDVI_Gm13.2* was identified in the single-year analyses. Four of the nine GNDVI QTLs identified in the combined analysis overlapped with NDVI QTL. The PVE of these GNDVI QTLs ranged from 2–13.7%, with the largest on Chr 12 (*qGNDVI_Gm12*). The PI 603535 allele was associated with increased NDVI and GNDVI for the QTLs identified in the combined analysis. The combined analysis identified only two NDRE QTLs that accounted for 6.1–7% PVE.

### Comparison between canopy wilting and remote sensing QTLs

In the combined analysis, CW QTLs colocalized with remote sensing QTLs on Chr 7 (*qWilt_Gm07*), 12 (*qWilt_Gm12*), and 13 (*qWilt_Gm13*) (Table 2, Supplemental Fig. 1). All three colocalized with NDVI QTLs, while *qWilt_Gm07* also colocalized with a GDNVI QTL. *qWilt_Gm12* and *qWilt_Gm13* accounted for the largest PVE and colocalized with NDVI QTL that accounted for the third and second largest PVEs, respectively. *qWilt_Gm07* accounted for a low PVE (3.4%) and colocalized with NDVI and GNDVI QTLs accounting for similarly low PVE. In the 2023 analysis, the Chr 7 CW QTL colocalized with the largest GNDVI QTL and second largest NDVI QTL by PVE. In the 2024 analysis, the largest CW QTL on Chr 12 colocalized with the largest NDVI and GNDVI QTLs.

## Discussion

### Variation of visual ratings

Benning × PI 603535-derived RILs exhibited substantial variation for CW scores in the combined analysis, indicating that certain lines may be useful for future drought tolerance breeding efforts and genetic studies. PI 416937 is a slow CW exotic Japanese accession that has been used extensively in breeding efforts in the southeastern USA both to broaden the genetic base and improve drought tolerance. Several high-yielding cultivars have been released in which PI 416937 accounts for between 12.5 and 50% of the pedigree (Bagherzadi et al. 2022; Boerma et al. 2012; Carter et al. 2003, 2007, 2008, 2016; Fallen et al. 2023). PI 603535’s CW score of 5.7 across years was substantially lower than that of PI 416937 (34.6). Steketee et al. (2020) found that PI 416937 exhibited a much lower CW score (18) than what was observed in the present study. Additionally, PI 603535 and 77 other genotypes exhibited lower CW scores across environments in the aforementioned study. In the present study, 122 RILs exhibited lower CW scores than PI 416937 across environments, five of which exhibited CW scores below 20. These results indicate that PI 603535 is a promising source of genetic variation for drought tolerance and may also have utility for developing improved soybean cultivars for drought tolerance. PI 603535 originated from the Shaanxi province of China, which is a semi-arid environment (USDA-ARS 2025). The exact physiological mechanisms that contribute to the slow CW phenotype of PI 603535 have not yet been characterized. A separate preliminary investigation evaluated Benning, PI 603535, and two RILs at each CW extreme under a rainout shelter and adjacent irrigated field, indicating that the slow CW genotypes exhibited more fibrous rooting systems than the fast CW genotypes under drought stress (unpublished results). These genotypes are also being evaluated in a gene expression and metabolite analysis to further elucidate the molecular mechanism and physiology of the slow CW phenotype.

### Heritability, correlations, and usefulness of remote sensing in drought phenotyping

CW broad-sense heritability across environments was high, *H*^*2*^ = 0.70 (Supplemental Table 1). This heritability is on the higher end of CW heritabilities found in previous studies which ranged from 0.34 to 0.80 (Abdel-Haleem et al. 2012; Charlson et al. 2009; Hwang et al. 2015; Kaler et al. 2017; Steketee et al. 2020; Zhou et al. 2020). In contrast, the remote sensing traits exhibited moderate to high heritabilities in the combined analyses (*H*^*2*^ = 0.49–0.68), with GNDVI and NDVI being the highest and most similar to CW. NDVI and nCT were the only remote sensing traits collected all three years and exhibited the highest single-year heritabilities in 2022. This result is somewhat surprising because remote sensing traits were collected with handheld devices in 2022, which requires longer phenotyping time and is more prone to measurement inconsistencies than UAV imagery. These factors were expected to increase the error variance, thus reducing the heritability. Despite these differences, there were no major outliers among any of the individual years for the remote sensing traits. The heritabilities for nCT have been previously found to be highly variable between environments, ranging between 0.07 and 0.62 (Bazzer & Purcell 2020; Kaler et al. 2018; Zhou et al. 2020). This is likely because CT only measures infrared radiation, whereas the multispectral VIs are normalized ratios of multiple wavelength intensities and are therefore more robust to fluctuations in the raw values due to environmental variation. Zhou et al. (2020) also found that NDVI and GNDVI exhibited the highest heritabilities among the remote sensing traits evaluated at 0.67 and 0.81, respectively.

GNDVI and NDVI were the only remote sensing traits to be significantly correlated with CW across-years (Fig. 3). GNDVI and NDVI are popular VIs for assessing plant health, with NDVI more sensitive to changes in vegetative cover and GNDVI more sensitive to changes in chlorophyll (Candiago et al. 2015; Gitelson et al. 1996; Rouse et al. 1974; Yoder & Waring 1994). In the present study, lower NDVI and GNDVI values were associated with higher CW scores. This is likely attributable to a combination of reduced canopy densities and greater exposure of the light green abaxial leaf surfaces due to leaf flipping (Campbell et al. 2007). The NDVI and GNDVI correlations with CW varied among individual years. The strongest correlations were observed in 2022 and 2023, whereas 2024 saw the weakest single-year correlations for all remote sensing traits. Surprisingly, there have been limited studies exploring the relationship between CW phenotype and multispectral remote sensing traits such as NDVI and GNDVI in soybean. Basal & Szabo (2020) found that increased drought stress reduced NDVI values across different nitrogen application treatments. Zhou et al. (2020) did not explicitly report the correlation between wilting scores and remote sensing traits, only that the NDVI of fast wilting lines was significantly lower than slow wilting lines. GNDVI was reported to be not significantly different between wilting classes. Burner et al., (2025, submitted) also found that NDVI and GNDVI exhibited consistently moderate-to-high correlations with CW. In the same study, NDRE consistently exhibited the weakest correlation with CW.

Canopy cover was noticeably lower in 2024 compared to 2023 based on aerial imagery, which likely explains the lower NDVI, GNDVI, and NDRE values (Fig. 2). Therefore, it is possible that the lower canopy density contributed to the reduced correlations. NDRE uses the red-edge band which penetrates deeper into the canopy because it is less strongly absorbed by foliar tissue (Barnes et al. 2000). This provides greater insight into overall canopy health and density, whereas GNDVI and NDVI mainly measure the topmost part of the canopy. In the present and previous studies, NDRE has consistently shown a weak correlation with CW (Poudel et al. 2023). This suggests that canopy density is weakly correlated with CW compared to the traits measured by NDVI and GNDVI in the topmost layer. Reduced canopy density undoubtedly exposes more leaves below the topmost canopy layer, therefore possibly contributing to the reduced GNDVI and NDVI correlations.

The correlation between nCT and CW varied greatly between years resulting in a non-significant correlation in either combined analysis (Fig. 3). In general, previous studies have found that slower wilting lines are generally associated with lower CT (Bai & Purcell 2018; Kaler et al. 2018; Zhou et al. 2020; Burner et al. 2025). Bai & Purcell (2018) found that slow wilting lines exhibited lower CTs than fast wilting lines, with the differences being significant as drought stress increased. Kaler et al. (2018) found a weak correlation (*R* = 0.25) between nCT and CW. As with NDVI and GNDVI, Zhou et al. (2020) did not report the correlation between CT and wilting scores but did find that fast wilting genotypes exhibited significantly higher CT than slow wilting genotypes. Lastly, Burner et al., (2025, submitted) also found that the strength of the nCT correlation with CW varied among individual environments, ranging from 0.10 to 0.60 in individual environments. The lower nCT of slow CW lines has been attributed to the cooling effect of a relatively higher transpiration rate (Jones et al. 2009). As described previously, CT is likely more influenced by environmental conditions than the multispectral VIs. The reduced nCT correlations in 2022 and 2024 compared to 2023 are possibly attributable to the use of a handheld device in the former and the reduced canopy densities in the latter. The reduced canopies, as with NDVI and GNDVI, may reduce the utility of CT as a proxy trait for CW.

Overall, the high heritabilities and correlations and low CVs for GNDVI and NDVI in the combined analyses indicate that these traits may be useful proxy traits for evaluating drought tolerance. Although CT has seen greater focus in previous drought research, the results of the present study indicate that its correlation with CW is variable, likely due to greater sensitivities to environmental changes during the course of a flight (wind, sunlight, ambient temperature, etc.). The lower resolution of thermal UAV cameras necessitates longer flights to capture a field during which time greater environmental variance can be introduced. Flight time can be reduced by increasing altitude thus increasing the footprint, but at the expense of reduced ground sampling distance and radiometric precision. UAV multispectral sensors typically have higher resolutions than thermal cameras, resulting in reduced flight times due to less image overlap needed. Additionally, the multispectral wavelengths are less influenced by environmental conditions than thermal data is. For these reasons, multispectral imagery is a more attractive option for supplementing drought studies not only because of its utility as a proxy trait but also because of the reduced logistical challenges. Further, unlike thermal imagery, the multiple bands of multispectral cameras offer a wider range of VIs to be extracted. Despite the utility of some multispectral remote sensing traits, we caution against relying on single-year measurements due to the evidence that the utility of these remote sensing traits can be greatly influenced by environmental factors.

### Comparisons to previous studies

This study identified seven unique QTLs associated with CW from the combined analysis across six different chromosomes (Table 2, Supplemental Fig. 1). Only two of these QTLs were identified in multiple individual years, indicating that the slow CW phenotype of PI 603535 is a highly polygenic trait controlled by QTLs with unstable effects across environments. The PI 603535 allele was considered favorable for six of the seven QTL, exhibiting negative additive effects (lower CW score). The genetic complexity of CW has been consistently observed in previous mapping and GWAS studies (Abdel-Haleem et al. 2012; Charlson et al. 2009; Hwang et al. 2015, 2016; Kaler et al. 2017; Menke et al. 2024; Steketee et al. 2020). Of the significant CW QTLs from the combined analysis, the putative locations of *qWilt_Gm02.1*, *qWilt_Gm02.2*, *qWilt_Gm12, qWilt_Gm13, qWilt_Gm19* were near or overlapped with major QTLs identified in previous studies. *qWilt_Gm02.1* is located within the intervals of the meta-QTLs *mqCanopywilt-001* and *mqCanopywilt-002* identified by Hwang et al. (2016). *qWilt_Gm02.2* is approximately 4.5 Mbp downstream of these meta-QTLs, in addition to being 1 Mbp upstream of Satt_351 identified as significant by Charlson et al. (2009). Further, *qWilt_Gm02.1* is 1 Mbp upstream of the SNP with the greatest absolute allelic effect identified in a genome-wide association study by Steketee et al. (2020). *qWilt_Gm12* accounted for the largest PVE in the present study, but the only other instance of this region being identified in another study was a SNP located 500 kbp upstream identified by Kaler et al. (2017) in a GWAS of MG IV accessions. *qWilt_Gm13* is located 5 Mbp downstream of Satt_362 identified by Charlson et al. (2009). *qWilt_Gm19* is located only 50 kbp downstream of a SNP identified by Steketee et al. (2020). Lastly, to our knowledge, this is the first drought mapping study in soybean to identify a major QTL on Chr 3 in an across-year analysis.

Prior to this study, CT is the only remote sensing trait that has been used as a remote sensing phenotype in previous drought mapping studies. In the present study, three nCT QTLs on separate chromosomes were identified in the combined analysis, none of which colocalized with CW QTLs. *qNCT_Gm07* is located 300 kbp upstream of a SNP identified in a single environment in an association mapping study performed by Kaler et al. (2018). *qNCT_Gm11* is located approximately 9 Mbp upstream of a Chr 11 SNP identified from a KS4895 × Jackson mapping study (Bazzer & Purcell 2020). *qNCT_Gm11* accounted for the highest PVE in both the present study (8.8%) and Bazzer & Purcell (2020) study (8.8%).

### Utility of remote sensing for drought tolerance QTL mapping

The two largest CW QTLs (*qWilt_Gm12* and *qWilt_Gm13*) by PVE colocalized with NDVI QTLs accounting for the second and third largest PVEs. The only other instance of colocalization in the combined analysis was between minor NDVI and GNDVI QTLs (PVE ≤ 3%) and a minor CW QTL (3.4%). The tendency for major CW and remote sensing QTLs to colocalize was inconsistent within individual years. In 2023, a Chr 7 CW QTL was the only CW QTL to colocalize with remote sensing QTL. In this case, the Chr 7 CW QTL accounted for a very low PVE (3%), whereas the colocalized NDVI and GNDVI QTLs accounted for much larger PVEs (> 8%). The largest 2023 CW QTL (12.6%) on Chr 13 was slightly upstream of the largest NDVI QTL (8.9%) and a minor GNDVI QTL (5.5%). In 2024, the largest CW QTL (13.6%) on Chr 12 colocalized with the largest NDVI and GNDVI QTLs. The 2024 Chr 13 CW QTL accounted for a low PVE (3.3%) but colocalized with the second largest NDVI QTL and second lowest GNDVI QTL.

These results indicate that certain remote sensing traits like NDVI and GNDVI are strong candidates as proxy traits for identifying the major genomic regions associated with CW. However, it is recommended to use remote sensing data from across multiple years due to the inconsistency. NDRE and nCT were not useful phenotypes for identifying the major genomic regions associated with CW. In this study, there was not a single instance of colocalization between CW and NDRE or nCT in any analysis. This result is unsurprising given the weak and variable correlations between CW and NDRE and nCT. Overall, the mapping results of both CW and the remote sensing traits further illustrate the genetic complexity of drought tolerance and highlights the need for multiple year evaluations to gain proper insight.

### Candidate genes

Candidate genes associated with plant stress or drought tolerance were identified within 10 kb of each of the selected QTLs (Table 3). *qWilt_Gm07* was located near *Glyma.07G202300* and *Glyma.07G202401* which encode cytochrome P450 (CYP) family proteins. CYPs are critical in the hormone signaling and the regulation of plant stress responses (Pandian et al. 2020). In soybean, CYP genes have been found to be upregulated due to drought and salt stress (Yan et al. 2016). *Glyma.07G202300* specifically was found to increase under salt stress and is associated with increased isoflavone accumulation, relative water content, root length, and root fresh weight (Jia et al. 2017).

**Table 3:** Candidate genes identified within 10 kbp of the Glyma.Wm82.a4 physical position of selected SNPs identified in the combined-year QTL analysis

QTL	SNP	Gene NAME	Annotation	References
*qWilt_Gm07*	Gm07_37260926	*Glyma.07G202300*	Cytochrome P450 family protein	Yan et al. (2016); Jia et al. (2017); Pandian et al.(2020)
		*Glyma.07G202401*		
*qWilt_Gm12*	Gm12_35626081	*Glyma.12G194200*	Glutamate receptor	Li et al. (2023)
		*Glyma.12G194300*	Hexokinase-1 protein	Wang et al. (2017); Jiao et al. (2023)
		*Glyma.12G194400*	Homeobox-leucine zipper protein	Cao et al. (2016)
*qWilt_Gm13*	Gm13_37988956	*Glyma.13G291400*	Basic helix-loop-helix (bHLH) domain	Zhang et al. (2022)
		*Glyma.13G291500*	Adaptin ear-binding coat-associated protein	Zhao et al. (2018)
*qNCT_Gm17*	Gm17_15728938	*Glyma.17G167700*	Cyclin D3	Nguyen et al*.* (2021)
*qNDVI_Gm13.1*	Gm13_579178	*Glyma.13G097600*	Kelch domain-containing protein	Wang et al. (2021); Wei et al. (2021)
*qGNDVI_Gm12*	Gm12_34102452	*Glyma.12G180067*	ARM repeat superfamily protein	Kojima et al. (2013);
		*Glyma.12G180133*		
		*Glyma.12G180200*	GDSL-like lipase/acylhydrolase superfamily protein	Su et al. (2020); Xu et al. (2022)
*qNDRE_Gm02*	Gm02_4159788	*Glyma.02G045800*	MID1-Complementing Activity 1-like isoform	Yadav et al. (2022)

The QTL with the largest PVE for each trait and CW QTL that colocalized with remote sensing QTL were selected

The gene models *Glyma.12G194200*, *Glyma.12G194300*, and *Glyma.12G194400* are located in the vicinity of *qWilt_Gm12. Glyma.12G194200* is annotated as a glutamate receptor. Li et al. (2023) found that certain glutamate receptor genes in soybean were upregulated in response to salt and jasmonic acid treatment indicating a role in stress signaling. *Glyma.12G194300* encodes a hexokinase-1 protein, which is a type of protein that transmits signals to regulate gene expression in response to sugar repression, such as from reduced photosynthesis (Jang & Sheen 1994). Wang et al. (2017) found that hexokinase levels were elevated in soybean roots shortly after flooding stress. Overexpression of hexokinases in soybean hairy roots has been found to improve tolerance to alkali stress (Jiao et al. 2023). *Glyma.12G194400* encodes a homeobox-leucine zipper protein which has been found to be induced by alkaline stress in the leaves and roots of *G. soja* and confer greater alkaline stress tolerance when overexpressed (Cao et al. 2016).

*qWilt_Gm13* was near *Glyma.13G291400* and *Glyma.13G291500*. *Glyma.13G291400* has a basic helix-loop-helix domain transcription factor annotation, which is a large family of transcription factors which are involved in a broad range of developmental and adaptive responses in plants (Hudson & Hudson 2015). *Glyma.13G291400* exhibited increased expression in leaf tissue in response to salt stress, suggesting a role in abiotic stress response (Zhang et al., 2022). *Glyma.13G291500* encodes an adaptin ear-binding coat-associated protein which has been associated with heat-stress response (Zhao et al. 2018).

*Glyma.17G167700* was the only gene model near *qNCT_Gm17* and has a cyclin D3 annotation which play roles in cell differentiation and proliferation in soybean (Nguyen et al. 2021). *Glyma.13G097600*, associated with *qNDVI_Gm13.1*, encodes a Kelch domain-containing protein, which has been previously associated with protein degradation in response to carbon degradation (Wang et al. 2021). *qGNDVI_GM12* was associated with three gene models: *Glyma.12G180067*, *Glyma.12G180133*, and *Glyma.12G180200*. *Glyma.12G180067* encodes an armadillo repeat superfamily protein that is involved in protein degradation, abscisic acid signaling, light, and gibberellin signaling (Kojima et al., 2013). *Glyma.12G180200* is annotated as a GDSL-like lipase/acylhydrolase superfamily protein which regulates plant growth, development, and stress signaling by hydrolyzing broad types of substrates (Su et al. 2020; Xu et al. 2022). Finally, *qNDRE_Gm02* is near the gene model *Glyma.02G045800* which encodes a MID1-Complementing Activity 1-like isoform, which helps facilitate the movement of Ca^2+^ during signal transduction in response to herbivore damage (Yadav et al. 2022). These candidate genes could serve as the focus of future studies to validate and characterize expression levels under drought stress.

The SNP genotypes associated with the peaks of each selected QTL were evaluated in the ten highest and lowest RILs by CW score (Table 1). PI 603535 contributed the favorable allele for all selected QTL. The 10 slowest wilting RILs were homozygous for the PI 603535 allele at 1–6 SNPs, whereas the 10 fastest wilting lines possessed the PI 603535 genotype at 0–5 SNPs. The PI 603535 genotype for *qWilt_Gm12* and *qGNDVI_12* was present in nine of the 10 slowest CW RILs, but only present in two of the fastest wilting RILs. This indicates that these markers could be used for marker-assisted selection or in genomic selection models to select for more drought-tolerant soybean genotypes (Miller et al. 2023). However, there are likely many underlying complex gene interactions influencing the CW phenotype that cannot be properly selected for using a handful of markers. Further, selecting based on the favorable genotypes at QTLs identified in this study may only result in a marginal improvement to CW due to the small additive effect estimates. This is further complicated by evidence that even the major QTLs are not consistently identified across environments. Regardless, the slow CW RILs likely possess genetic variation that is unaccounted for by the QTL models used in the present study but can nonetheless be inherited and selected for.

## Conclusion

A Benning × PI 603535 RIL population was used to identify seven CW QTLs, including the first major CW QTL on Chr 3. The multispectral VIs GNDVI and NDVI exhibited moderate correlations with CW scores across years; however, there were variations in correlation among individual years. Normalized CT exhibited a low correlation with CW scores likely due to greater influence of the environment. These results indicate that remote sensing can be deployed in breeding programs to evaluate drought tolerance. QTLs for CW and remote sensing traits were inconsistently identified across years; however, there was a tendency for remote sensing QTLs to colocalize with the major CW QTLs, indicating some utility for using remote sensing to identify underlying genetic variation. The slow CW RILs developed during this study can serve as breeding stock for genetic improvement of drought tolerance and genetic studies. This study highlights the genetic complexity of soybean drought tolerance and demonstrates the utility of deploying remote sensing to phenotype drought tolerance.

## Supplementary Information

Below is the link to the electronic supplementary material.

Supplementary file1 (DOCX 2551 KB)

## Data Availability

The data set generated from this study is available from the corresponding author upon request.
