# Supplementary material for: Identifying canopy wilting QTLs and evaluating remote sensing approaches for selecting drought-tolerant soybean
Source: Theor Appl Genet. 2025 Oct 14;138(11):276. doi: 10.1007/s00122-025-05063-y (PMC12521297; doi:10.1007/s00122-025-05063-y)
Supplement: Supplementary file 1 — Supplementary file1 (DOCX 2551 KB) [file 122_2025_5063_MOESM1_ESM.docx]

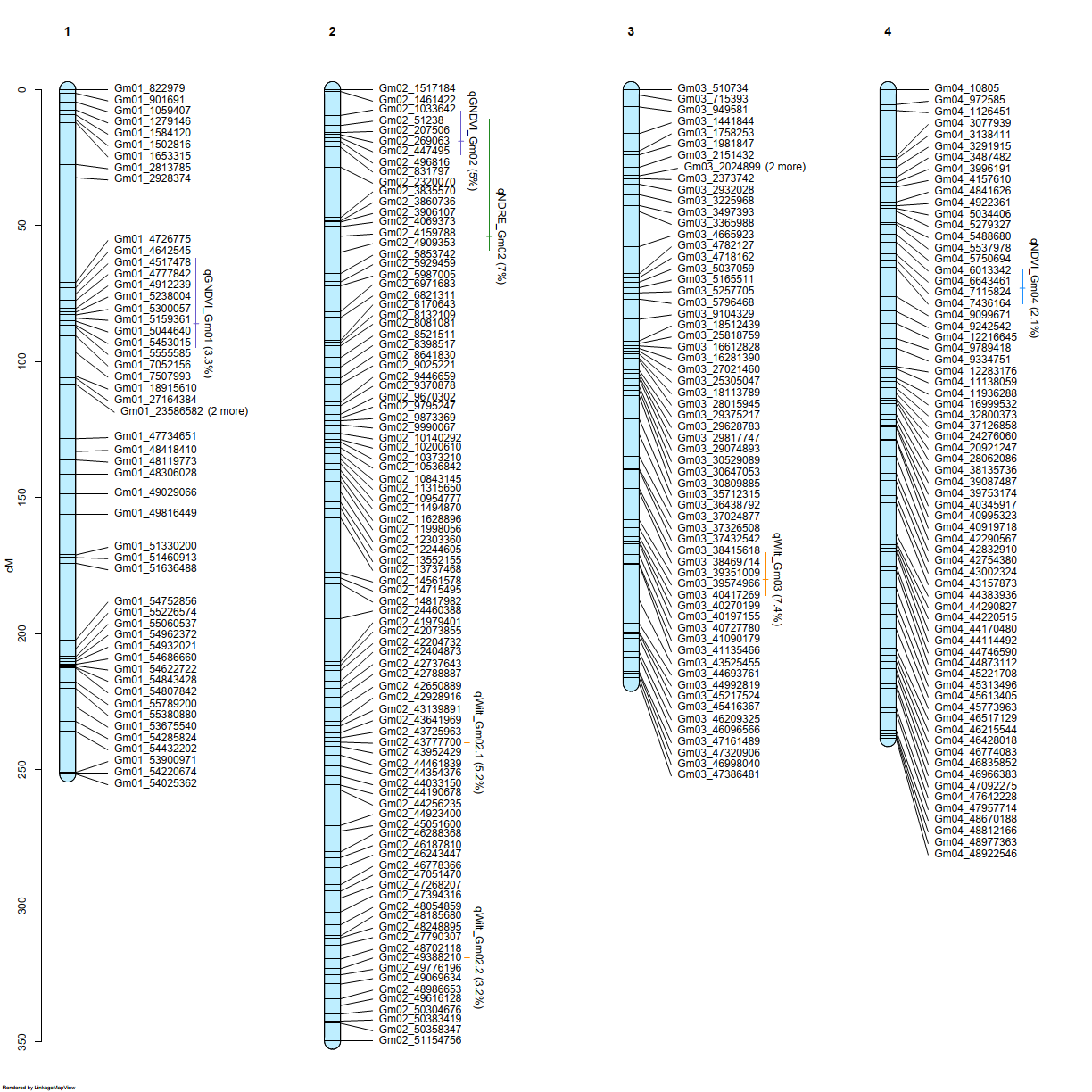


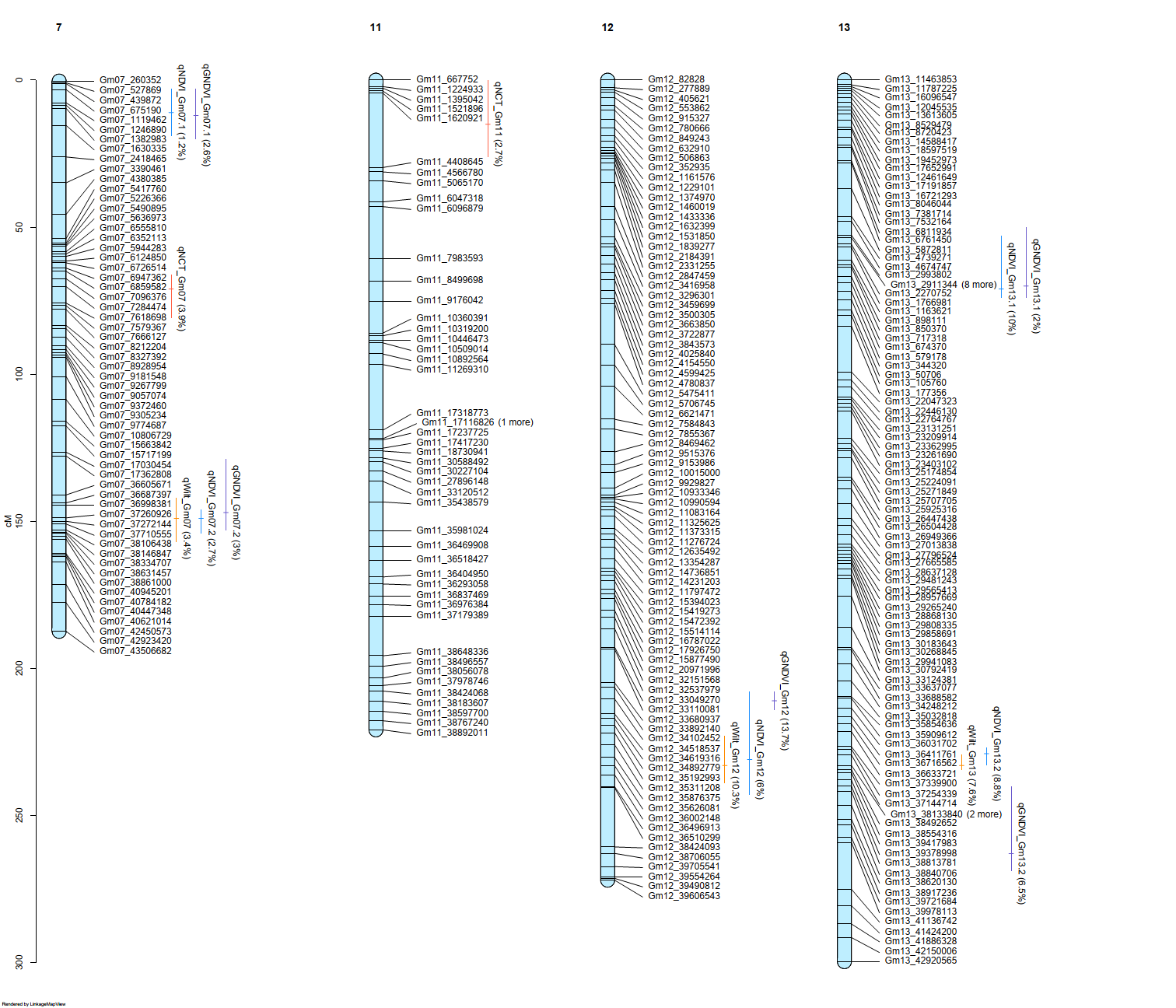


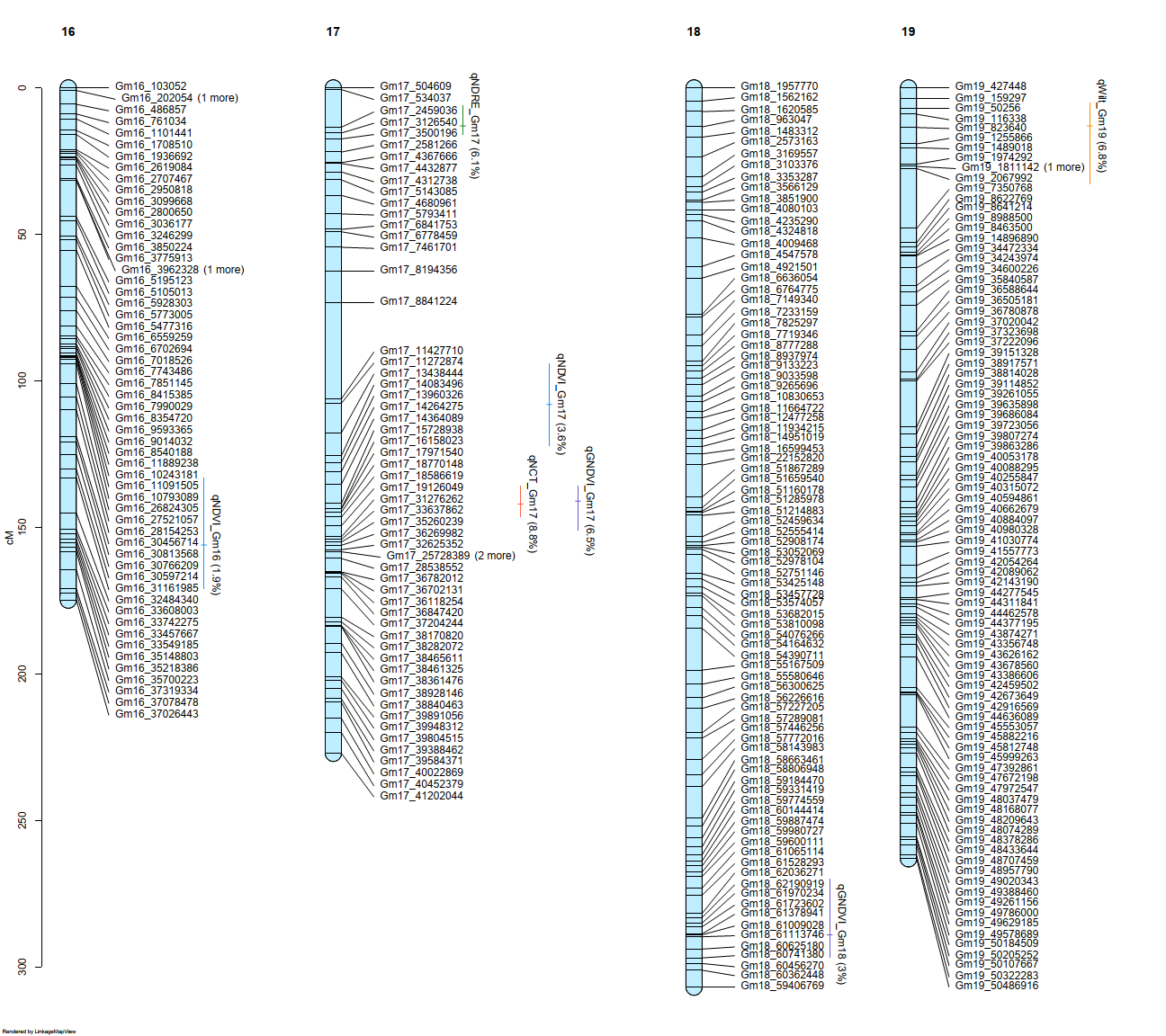


**Supplemental Figure 1:** Genetic map with 1.0-LOD confidence interval for the significant QTL identified in combined analysis. Only chromosomes with identified QTL are shown.

**Supplemental Table 1:** Analysis of variance results for CW and remote sensing traits in combined and single environments.

|  |  |  | **CW** | | | **nCT** | | | **NDVI** | | | **GNDVI** | | | **NDRE** | | |
| --- | --- | --- | --- | --- | --- | --- | --- | --- | --- | --- | --- | --- | --- | --- | --- | --- | --- |
| **Year** | **Source^a^** | **DF** | **MS** |  | **H^2^** | **MS** |  | **H^2^** | **MS** |  | **H^2^** | **MS** |  | **H^2^** | **MS** |  | **H^2^** |
| 3-years | Y | 2 | 42305.4 | *** | 0.70 | 3.5856 | *** | 0.49 | 2.7862 | *** | 0.62 |  |  |  |  |  |  |
|  | G^b^ | 194 | 379.4 | *** |  | 0.0299 |  |  | 0.0019 | *** |  |  |  |  |  |  |  |
|  | G × Y | 388 | 138.5 | ** |  | 0.0261 |  |  | 0.0011 | *** |  |  |  |  |  |  |  |
|  |  |  |  |  |  |  |  |  |  |  |  |  |  |  |  |  |  |
| 2-years^c^ | Y | 1 | 9219.7 | *** | 0.68 | 6.0091 | *** | 0.54 | 5.3545 | *** | 0.65 | 5.3117 | *** | 0.68 | 4.6851 | *** | 0.5 |
|  | G^b^ | 194 | 272.0 | *** |  | 0.0302 | * |  | 0.0014 | *** |  | 0.0015 | *** |  | 0.0012 |  |  |
|  | G × Y | 194 | 134.1 | ** |  | 0.0238 |  |  | 0.0008 | * |  | 0.0007 | ** |  | 0.0011 | ** |  |
|  |  |  |  |  |  |  |  |  |  |  |  |  |  |  |  |  |  |
| 2022 | G | 194 | 241.2 | ** | 0.61 | 0.0282 |  | 0.54 | 0.0018 | ** | 0.60 |  |  |  |  |  |  |
|  |  |  |  |  |  |  |  |  |  |  |  |  |  |  |  |  |  |
| 2023 | G | 194 | 281.1 | *** | 0.56 | 0.0311 |  | 0.35 | 0.0007 | *** | 0.49 | 0.0008 | *** | 0.52 | 0.0013 | ** | 0.41 |
|  |  |  |  |  |  |  |  |  |  |  |  |  |  |  |  |  |  |
| 2024 | G | 194 | 132.9 | *** | 0.50 | 0.0236 | *** | 0.49 | 0.0014 | *** | 0.49 | 0.0013 | *** | 0.51 | 0.0010 | ** | 0.47 |
| F-value significance indicated as *** < 0.001, ** < 0.01, * < 0.05  CW= Canopy wilting; nCT = Normorlized canopy temperature; NDVI = Normalized difference vegetation index; GNDVI = Green-based normalized difference vegetation index; NDRE = Normalized difference red edge index | | | | | | | | | | | | | | | | | |
| ^a^ Source of variance. Y = year, G = genotype | | | | | | | | | | | | | | | | | |
| ^b^ G × Y MS was used as the denominator for significance testing | | | | | | | | | | | | | | | | | |
| ^c^ 2023-2024 used the same UAV for remote sensing traits whereas 2022 used a handheld device. Separate analyses are done to reflect this difference | | | | | | | | | | | | | | | | | |

**Supplemental Table 2:** CW and remote sensing QTL identified by QTL mapping using BLUPs as phenotype values from individual year analyses.

| **Year** | **Trait** | **Chr^a^** | **Nearest SNP** | **Pos (bp)^b^** | **Pos (cM)^c^** | **CI (cM)^d^** | **LOD^e^** | **PVE^f^** | **Effect^g^** | **+ Allele^h^** |
| --- | --- | --- | --- | --- | --- | --- | --- | --- | --- | --- |
| 2022 | CW | 12 | Gm12_35626081 | 37,053,933 | 233 | 210-235 | 4.7 | 10.7 | -1.23 | PI |
|  |  |  |  |  |  |  |  |  |  |  |
| 2023 | CW | 1 | Gm01_7507993 | 7,518,008 | 99 | 86.6-108.3 | 3.3 | 1.6 | -0.74 | PI |
|  |  | 2 | Gm02_44033150 | 42,789,067 | 251 | 223.3-267 | 3.4 | 2.4 | -0.92 | PI |
|  |  | 3 | Gm03_40727780 | 39,888,526 | 171 | 168-186 | 6.7 | 8.8 | -1.72 | PI |
|  |  | 6 | Gm06_3608127 | 3,621,588 | 39 | 23-44.7 | 3.6 | 1.3 | 0.66 | B |
|  |  | **7** | **Gm07_37260926** | **37,488,675** | **149** | **144.2-158** | **3.1** | **3.0** | **-1.00** | **PI** |
|  |  | 12 | Gm12_34102452 | 35,530,221 | 209 | 191-223 | 3.0 | 4.5 | -1.29 | PI |
|  |  | 13 | Gm13_37988956 | 38,538,394 | 233 | 232-234.4 | 12.3 | 12.6 | -2.04 | PI |
|  |  | 19 | Gm19_823640 | 855,537 | 16 | 10-26 | 4.2 | 2.1 | -0.86 | PI |
|  |  |  |  |  |  |  |  |  |  |  |
|  | nCT | 17 | Gm17_14083496 | 13,782,545 | 126 | 120-143.5 | 3.0 | 6.3 | -1.99E-03 | PI |
|  |  |  |  |  |  |  |  |  |  |  |
|  | NDVI | 4 | Gm04_37126858 | 39,106,913 | 112 | 108-115 | 4.1 | 1.8 | -9.41E-04 | B |
|  |  | **7** | **Gm07_37272144** | **37,499,885** | **150** | **148.5-152.6** | **6.6** | **8.9** | **2.26E-03** | **PI** |
|  |  | 13 | Gm13_2993802 | 17,901,116 | 52 | 49-54 | 3.5 | 0.9 | 7.42E-04 | PI |
|  |  | 13 | Gm13_39978113 | 40,528,851 | 263 | 237-272 | 6.1 | 9.8 | 2.34E-03 | PI |
|  |  | 17 | Gm17_2459036 | 2,451,384 | 9 | 0-19 | 3.0 | 3.0 | -1.32E-03 | B |
|  |  |  |  |  |  |  |  |  |  |  |
|  | GNDVI | **7** | **Gm07_37260926** | **37,488,675** | **149** | **146-163** | **5.7** | **8.2** | **2.60E-03** | **PI** |
|  |  | 13 | Gm13_39978113 | 40,528,851 | 267 | 235-280 | 3.8 | 5.5 | 2.00E-03 | PI |
|  |  | 17 | Gm17_14083496 | 13,782,545 | 126 | 120-131 | 3.7 | 4.2 | 1.87E-03 | PI |
|  |  | 18 | Gm18_61528293 | 57,502,699 | 278 | 263.8-289.7 | 3.5 | 5.9 | 2.22E-03 | PI |
|  |  |  |  |  |  |  |  |  |  |  |
| 2024 | CW | 2 | Gm02_43641969 | 42,397,487 | 236 | 233-242 | 3.1 | 4.5 | -0.52 | PI |
|  |  | **12** | **Gm12_36496913** | **37,912,810** | **240** | **228-246** | **7.8** | **13.6** | **-0.89** | **PI** |
|  |  | **13** | **Gm13_37988956** | **38,538,394** | **233** | **230-237** | **3.2** | **3.3** | **-0.44** | **PI** |
|  |  |  |  |  |  |  |  |  |  |  |
|  | nCT | 2 | Gm02_41979401 | 40,738,291 | 205 | 194.4-212 | 3.0 | 2.8 | 5.73E-03 | B |
|  |  | 17 | Gm17_15728938 | 15,449,724 | 142 | 139-153 | 7.2 | 9.7 | -1.18E-02 | PI |
|  |  | 17 | Gm17_38840463 | 38,476,940 | 196 | 190-201 | 3.3 | 2.6 | 6.78E-03 | B |
|  |  | 20 | Gm20_14323433 | 10,195,563 | 61 | 52-76 | 3.3 | 4.6 | 7.69E-03 | B |
|  |  |  |  |  |  |  |  |  |  |  |
|  | NDVI | 8 | Gm08_34506326 | 34,530,763 | 57 | 52-87 | 4.4 | 3.4 | 1.52E-03 | PI |
|  |  | 9 | Gm09_43874852 | 47,464,029 | 193 | 180-201 | 3.3 | 3.2 | -1.44E-03 | B |
|  |  | **12** | **Gm12_35192993** | **36,621,521** | **222** | **213-243** | **4.8** | **8.5** | **2.38E-03** | **PI** |
|  |  | **13** | **Gm13_37144714** | **37,687,029** | **230** | **216.4-234** | **5.2** | **5.7** | **1.99E-03** | **PI** |
|  |  | 17 | Gm17_5793411 | 5,528,330 | 41 | 25.5-49 | 4.2 | 5.7 | -1.99E-03 | B |
|  |  |  |  |  |  |  |  |  |  |  |
|  | GNDVI | 2 | Gm02_496816 | 496,539 | 19 | 10-41 | 4.4 | 5.0 | 2.02E-03 | PI |
|  |  | 8 | Gm08_34506326 | 34,530,763 | 57 | 53-88 | 4.4 | 4.7 | 1.96E-03 | PI |
|  |  | 9 | Gm09_43874852 | 47,464,029 | 193 | 181.8-199 | 4.1 | 2.2 | -1.35E-03 | B |
|  |  | **12** | **Gm12_35192993** | **36,621,521** | **222** | **208-236** | **5.4** | **7.9** | **2.48E-03** | **PI** |
|  |  | **13** | **Gm13_37144714** | **37,687,029** | **231** | **215-253** | **5.2** | **3.5** | **1.68E-03** | **PI** |
|  |  |  |  |  |  |  |  |  |  |  |
|  | NDRE | 2 | Gm02_3835570 | 3,868,257 | 38 | 17-43 | 4.8 | 8.6 | 1.45E-03 | PI |
| Bold QTL indicate colocalization between CW and remote sensing QTL(s)  CW= Canopy wilting; nCT = Normorlized canopy temperature; NDVI = Normalized difference vegetation index; GNDVI = Green-based normalized difference vegetation index; NDRE = Normalized difference red edge index | | | | | | | | | | |
| ^a^ Chromosome | | | | | | | | | | |
| ^b^ Glyma.Wm82.a4 physical position in base pairs (Song et al., 2024) | | | | | | | | | | |
| ^c^ Genetic map position in centiMorgans | | | | | | | | | | |
| ^d^ 1.0-LOD confidence interval | | | | | | | | | | |
| ^e^ Log_10_ likelihood ratio comparing full model with reduced model (term dropped) | | | | | | | | | | |
| ^f^ Percent variation explained | | | | | | | | | | |
| ^g^ Additive allelic effect | | | | | | | | | | |
| ^h^ Source of the allele with favorable effect. B = Benning, PI = PI 603535. Negative effects for CW and nCT and positive effects for NDVI, GNDVI, and NDRE were considered favorable | | | | | | | | | | |
